# The MNV-1 protease–polymerase precursor cleaves a novel site in the NS1-2 protein

**DOI:** 10.1099/jgv.0.002263

**Published:** 2026-04-29

**Keywords:** murine norovirus, NS1-2, polyprotein processing, precursor protein, protease, protease–polymerase (ProPol)

## Abstract

Human noroviruses (HuNVs) are members of the *Caliciviridae* family and are a significant cause of gastroenteritis worldwide. Cell culture models are challenging for studying the intracellular replication of HuNV, with murine norovirus (MNV) routinely used to study norovirus replication. Norovirus genomes contain a non-structural (NS) polyprotein that is processed by the viral protease, generating precursor and mature proteins during replication. We identified a putative viral protease cleavage site at E^66^/G^67^ in the MNV-1 NS1-2 protein by sequence analysis that was cleaved by the protease–polymerase (ProPol) precursor but not Pro *in vitro* and during MNV-1 replication, with the cleavage site confirmed by MS. The P4-P4’ LHAE^66^/G^67^PLA cleavage site of MNV-1 is conserved in some MNV strains, while other strains contain a predicted caspase cleavage site (LHAD^66^/G^67^PHA) at this position, indicating that cleavage of NS1 is widely conserved in MNV. Mutation of the ProPol cleavage site in MNV-1 NS1-2 to the caspase motif reduced viral protein expression and replication in Huh7CD300lf and BV2 cells, while removal of the site via an E^66^A mutation caused a 100-fold reduction in viral yield in the naturally permissive BV-2 cell line. This study demonstrates that the protease function of the MNV-1 ProPol precursor has an important role during replication that is distinct to the mature protease and that cleavage of NS1-2 by ProPol at E^66^/G^67^ is important for MNV-1 replication.

**Impact Statement**

Viruses with +ssRNA genomes often express some or all of their proteins as a large polyprotein that is processed by viral protease(s) to mature functional proteins essential for virus replication. Viral polyprotein cleavage also generates a range of partially processed precursors with variable activity to the fully processed mature proteins, increasing the coding capacity of the viral genome and contributing to viral replication. One precursor in noroviruses is the protease–polymerase precursor, ProPol. The polymerase activity of ProPol varies to the mature Pol, but little is known about the protease activity of the ProPol precursor. We identified an apparent viral protease cleavage site in the NS1 region of the murine norovirus (MNV-1) NS1-2 protein and showed that this site is processed by the ProPol precursor but not the mature protease. Mutating the ProPol site in NS1 confirmed that this ProPol precursor activity and consequently the forms of NS1 or NS1-2 generated by ProPol during infection are critical for efficient MNV replication. NS1 is a highly disordered protein with multiple roles being slowly dissected. This research identifies a unique substrate recognition capability for ProPol compared to Pro and adds to the complexity of NS1-2 proteins generated during norovirus infection that impact upon viral replication.

## Data Summary

The authors confirm that all supporting data, code and protocols have been provided within the article.

## Introduction

Human noroviruses (HuNV; *Caliciviridae*) are one of the major causes of gastroenteritis globally [1]. The disease burden is due to the high rates of transmission and severity of disease with both direct and indirect costs estimated to be US$4.2 billion and ~US$603 billion, respectively [2]. Significant progress in culturing HuNV, including in transformed B-cells, stem cell-derived organoids and zebrafish larvae, has improved the understanding of HuNV replication [3]. Despite these advances, culturing HuNV remains challenging and expensive with no system able to sustain viral replication beyond limited passage of faecal inocula. Murine norovirus (MNV) is a commonly utilized model for HuNV replication due to similarities in genomic organization and virus structure [4].

The +ssRNA genome of MNV and HuNV is organized into four ORFs; ORF1 encodes the non-structural (NS) proteins, ORF2 and ORF3 encode the major and minor capsid proteins, respectively, and ORF4 encodes a virulence factor found only in MNV. The ORF1 polyprotein is translated during replication and subsequently processed by the viral protease (Pro) at specific cleavage sites to produce the mature NS proteins: NS1-2, NS3, NS4, NS5 (VPg), NS6 (Pro) and NS7 (Pol) [5,7].

Multifunctional proteins are a common strategy used to compensate for the limited coding potential of a small viral genome. Multiple proteins can be generated by additional protein processing by host or viral proteases, or the viral protease cleaving protease sites with different efficiencies, resulting in the accumulation of precursor proteins [8]. These precursors can have separate or additional functions to the mature proteins. One of the best described examples is the 3CD protein precursor of poliovirus that preferentially cleaves the P1 capsid protein and induces Arf protein translocation to cell membranes, neither of which occur with the mature 3C protease [9,10]. The protease–polymerase (ProPol) precursor from the calicivirus family is analogous to the poliovirus 3CD protein and has been observed in viruses from the *Lagovirus*, *Vesivirus* and *Norovirus* genera [11,14].

The ProPol precursor from HuNV is a multifunctional protein with activities distinct from the mature protease and polymerase [15,18]. HuNV ProPol displayed increased affinity for poly-U and poly-A RNA templates, with Pol unable to utilize poly-A as a template [17]. HuNV ProPol and Pol also display nucleotidylylation activity [19] with ProPol nucleotidylyating VPg 100-fold more efficiently than Pol [16].

Comparisons of the protease activity between HuNV ProPol and Pro demonstrated that ProPol cleaved substrates at similar levels to, or more efficiently than, Pro [15] which is proposed to contribute to the ORF1 cleavage site processing order. To date, there has been no *in vitro* data indicating if MNV ProPol has additional or separate functions to the mature protease.

The norovirus NS1-2 protein is partially cleaved by host caspases into NS1 and NS2 [6]. *In vitro* experiments identified that MNV NS1-2 was cleaved by caspase 3 at amino acids 121 and 128, while caspase 7 was shown to cleave HuNV NS1-2 [20]. Other NS1-2 protein fragments have been observed during MNV infection; however, the cause of these fragments is unknown [21,22]. Removal of the caspase cleavage sites in MNV reduced replication in the ilium by tenfold, showing that NS1-2 cleavage is important for viral replication [21]. NS1-2 fragments are also important for determining cell tropism with expression of NS1 from the CR6 MNV strain being important for replication in intestinal epithelial cells [23].

This work aimed to establish if the protease activity of MNV ProPol has a different role in viral replication to the mature protease by characterizing a previously undescribed protease site in the NS1-2 region of the ORF1 polyprotein of MNV-1.CW3. Our results confirm that MNV ProPol has increased protease activity on an established protease cleavage sequence compared to the mature Pro and that ProPol, but not Pro, cleaves an uncharacterized protease site in NS1-2. Finally, we have shown that altering the ProPol cleavage site in NS1-2 decreases replication *in vitro*. Overall, this work highlights that MNV-1.CW3 Pro has altered substrate recognition when in association with the polymerase (ProPol), generating processed forms of NS1-2 that are not made by the mature protease that are important to MNV-1.CW3 replication.

## Methods

### Cell lines

Murine macrophage RAW264.7 cells (ATCC; RRID:CVCL_0493), murine microglial BV-2 and Huh7CD300lf cells were gifts from Prof. Stefan Taube, University of Lübeck, Germany. The Huh7 cells stably express the CD300lf receptor for MNV infection [24].

### Cell culture

Each cell line was maintained at 37 °C with 5% CO_2_ and passaged at 70–80% confluency. RAW264.7 and BV-2 cells were cultured in Dulbecco’s modified Eagle’s medium (DMEM), 4 mM l-glutamine and 4.5 g l^−1^ glucose supplemented with 10% heat-inactivated FBS. The Huh7CD300lf cells were cultured in DMEM, 4 mM l-glutamine and 1 g l^−1^ glucose supplemented with 10% FBS, 1 µg ml^−1^ puromycin and 0.2 mg ml^−1^ G418.

### Preparation of MNV-1 stocks

MNV-1.CW3, originally generated through reverse genetics, described by Ward *et al*. [25], was propagated through RAW264.7 cells. The virus-infected cells were lysed by two freeze-thaw cycles, and the lysate was clarified by centrifugation at 10,000 ***g***. The virus was purified via ultracentrifugation at 112,700 ***g*** through a 30% (wt/vol) sucrose cushion. The pellet was resuspended in DMEM and filter-sterilized to make a purified virus stock. Virus stocks were quantified via crystal violet plaque assay [26,27].

Cells were infected with MNV at the indicated multiplicity of infection (MOI). The virus was incubated on the cells for 1 h and then removed, and fresh medium was added. The infected cells were incubated for the indicated times, and then, the cells were harvested for Western blot analysis to detect MNV proteins, and the supernatant was collected and quantified for viral titre by plaque assay.

### Detection of MNV-1 viral proteins

Mock-infected and MNV-1-infected cells were pelleted and then washed in sterile Dulbecco’s phosphate-buffered saline (dPBS). The cell pellets were resuspended in dPBS and 1x SDS-PAGE sample buffer and were stored at −20 °C until analysis. The samples were electrophoresed on an SDS-PAGE gel, and the proteins were transferred onto a reinforced nitrocellulose membrane (Amersham). Protein expression was detected with a primary and a corresponding secondary antibody: MNV-1 NS1-2 [22], MNV-1 VP1 [22], mouse alpha-tubulin (Ab 7291 and Ab18251; Abcam Limited), DyLight 800 donkey anti-rabbit IgG (SA5-10040; Thermo Scientific), DyLight 680 donkey anti-mouse IgG (SA5-10090; Thermo Scientific) and donkey anti-mouse IgG IRDye 800 CW (926-32212; LI-COR Biosciences).

For quantification of viral proteins, an additional MNV-1-infected sample was electrophoresed on a gel lane for use as a standard. Densitometry was performed using ImageStudio Lite software 5.2.5 (LI-COR Biosciences). Viral protein expression was normalized to the tubulin loading control and then expressed as relative to the MNV-1 standard.

### Caspase prediction

Residues 1–341 of NS1-2 of MNV-1.CW3 (DQ285629) and MNV-1.CR6 (EU004676) were sourced from GenBank, and caspase cleavage sites were predicted using ProsperousPlus software with default settings for caspases 3, 6, 7 and 8 [28].

### Cloning MNV-1.CW3 ProPol, Pro and ppdko

MNV ProPol contained a Q181G mutation to prevent processing at the Pro/Pol cleavage junction. To construct a catalytically inactive ProPol (ppdko), a synthetic gene (GenScript) was generated with a C139G mutation to remove protease activity plus D528G and D529G mutations to remove polymerase activity. PCR primers (Table 1) synthesized by Integrated DNA Technologies (IDT) were used to create homologous overhangs to clone MNV ProPol, ppdko and Pro into the pRham^™^ N-His SUMO (pRham) expression vector.

**Table 1.** Sequence of primers to construct the ProPol, Pro and ppdko expression vectors

Primer	Sequence (5′−3′)^*†^
SUMO.MNV.ProPol F	CGCGAACAGATAGGAGGTGCCCCAGTCTCCATCT
SUMO.MNV.ProPol R	**GTGGCGGCCGCTCTATTA**CTCATCCTCATTCACAAAG
SUMO.MNV Pro F	CGCGAACAGATAGGAGGTGCCCCAGTCTCCATCTGGTCA
SUMO.MNV Pro R	**GTGGCGGCCGCTCTATTA**CTGGAACTCCAGAGCCTCAA

*The underlined nucleotides indicate the complementary overhangs to the SUMO fusion tag.

† The bolded nucleotides indicate complementary sequences to the pRham vector.

The PCR products and linearized pRham vector were transformed together into *Escherichia cloni* 10G (Lucigen) competent *E. coli* per the manufacturer’s instructions. Recombination of the homologous ends of the PCR products and linear pRham vector in the E. cloni cells inserts the target gene into the vector to generate a circular vector. Clones were screened using restriction digests with EcoRI (Roche). Final plasmids were confirmed by whole-plasmid sequencing performed by Plasmidsaurus using Oxford Nanopore Technology.

### Expression and purification of *E. coli*-expressed proteins

The cultures of individual colonies were grown at 37 °C, 200 r.p.m. until the OD_600_ reached 0.5. l-Rhamnose solution was added to 0.2% to induce protein expression. The culture was incubated for 5 h at 37 °C, 200 r.p.m and harvested via centrifugation.

Cells were resuspended in 50 mM HEPES pH 7.5, 300 mM NaCl, 20% glycerol, 2 mM beta-mercaptoethanol (2-ME) and 25 mM imidazole buffer containing 1 mg ml^−1^ lysozyme and lysed via sonication. The proteins were purified with Ni-NTA resin (Qiagen) and eluted in the above buffer containing 120 mM imidazole. To remove the SUMO tag, the proteins were buffer-exchanged into 50 mM HEPES pH 7.5, 300 mM NaCl, 10% glycerol and 2 mM 2-ME and cleaved using His-tagged SUMO protease at 1 U mg^−1^ viral protein overnight at 4 °C. The SUMO protease and cleaved SUMO tag were removed using Ni-NTA resin. Proteins were buffer-exchanged using 3 kDa MWCO Amicon centrifugation filters (Pro) or by dialysis (10K MWCO) into 50 mM HEPES pH 8, 150 mM NaCl, 2 mM 2-ME and 50% glycerol (ProPol and ppdko). Protein quantification was determined via densitometry on SDS-PAGE gels using BSA (ProPol, ppdko) or lysozyme (Pro) as standards.

### Chemical synthesis

The peptide substrate [5(6)-carboxyfluorescein (FAM)-FGEWQAEGPFDALD-Dabcyl] representing the MNV-1 NS2/3 cleavage site was synthesized as described in [29]. The NS1c-2 cleavage site substrate Edans-LAALHAEGPLAGL-Dabcyl was purchased from JPT Technologies.

### Protease assay for MNV ProPol and Pro

The peptide substrate, 5(6)-carboxyfluorescein (FAM)-FGEWQA**EG**PFDALD-Dabcyl (bolded residues indicate the P1-P1' scissile bond), was used to assess protease activity. MNV ProPol or Pro was diluted to 0.25 µM in protease assay buffer (10 mM HEPES pH 8, 30% glycerol, 0.1% CHAPS and 2 mM DTT) and incubated with serial dilutions of the peptide substrate. The plate was mixed for 2 min (min) at 800 r.p.m. Fluorescent measurements were taken every minute for 30 min on a VarioSkan Lux multimode plate reader (Thermo Scientific) (ex/em=492/517). The substrate-only control for each concentration was subtracted, and the relative fluorescent unit values were corrected for the inner filter effect per [30] and converted to products using a FAM or Edans standard curve. The initial rates (0–12 min) were calculated for each progress curve and plotted against substrate concentration. The data were fitted to a Michaelis–Menten equation in GraphPad Prism v10 to generate Vmax, Km and kcat values.

### Identification of the NS1c-2 fragments

#### NS1-2.TM protein purification

MNV-1 (GenBank DQ285629) NS1-2 amino acids 3–260 without the C-terminal transmembrane region (NS1-2.TM; 29.1 kDa) was expressed and purified from *Trichoplusia ni* cells as described by Mirabelli *et al*. [20].

#### NS1-2.TM cleavage assay

NS1-2.TM was incubated with increasing amounts of ProPol and Pro for 4 h at 37 °C in reaction buffer containing 10 mM HEPES pH 8, 30% glycerol, 0.1% CHAPs and 2 mM DTT. Reactions were stopped with the addition of an equal volume of 2x SDS-PAGE sample buffer containing 8 M urea. The samples were visualized on 15% SDS-PAGE with Coomassie Brilliant Blue G250 stain.

To identify where cleavage of the NS1-2.TM occurred, the protein band corresponding to the 36 kDa fragment (NS1c-2) was excised from the gel, as was the full-length NS1-2.TM protein from the same lane. The samples were submitted to the Centre for Protein Research, University of Otago, for MS analysis [20]. Briefly, gel fractions were in-gel digested with trypsin or chymotrypsin. The resulting peptides were injected on an in-house packed emitter-tip column filled with Luna^™^ 3 µM C18 beads (Phenomenex) at a flow rate of 400 nl min^−1^ using an Ultimate 3000 nano uHPLC (ultra HPLC) system (Thermo Scientific) and separated by a gradient from 2% (v/v) acetonitrile (ACN), 0.05% (v/v) formic acid (FA) in water to 95% (v/v) ACN, 0.05% (v/v) FA in water over 45 min for data-dependent acquisition tandem MS (DDA-MS/MS) or 60 min for targeted parallel reaction monitoring (PRM). DDA-MS/MS was performed on an LTQ Orbitrap XL (Thermo Scientific) and PRM on an Orbitrap Exploris 240 (Thermo Scientific) mass spectrometer. For the PRM analysis, the tryptic peptide ^82^VLIFNEWEER^91^ ([M+2 h]^2+^=667.8406) being present in both NS1 and NS1c-2 and the semi-tryptic peptide ^67^GPLAGLPVTR^76^ ([M+2 h]^2+^=490.7980) specific for the N-terminus of the NS1c-2 fragment were targeted. Data analysis was performed using the Proteome Discoverer software (version 2.5, Thermo Scientific) for DDA-MS/MS data and the Skyline software for PRM data. Sequence coverage of the MNV NS1-2.TM fragment was compared to the sequence of the full-length control.

To identify the amino acid sequence of the NS1c-2 fragment from infected cells, BV-2 cells were infected at an MOI of 10. The infected sample was electrophoresed on an SDS-PAGE gel, and bands corresponding to the estimated sizes of NS1, NS2 and NS1c-2 were excised from the gel alongside purified NS1-2.TM as a control. The four fractions were processed as above.

### Identification of N-terminal peptides

The iTRAQ-TAILS methodology was performed as described by Kleifeld *et al*. [31]. RAW264.7 cells were grown to 80% confluency. The cells were centrifuged at 300 ***g*** for 5 min and washed with dPBS. The cells were lysed via sonication in 50 mM HEPES, 10% glycerol and 150 mM NaCl using the recommended settings for mammalian cells (Branson SFX250 Sonifier 250W Ultrasonic Cell Disrupter). The lysate was centrifuged at 5,000 ***g*** for 10 min.

MNV Pro, ProPol or ppdko was incubated with soluble RAW264.7 cell lysate at a ratio of 1 µg enzyme to 50 µg lysate in cleavage assay reaction buffer with 5 µg of NS1-2.TM added into each reaction. After the reactions were incubated at 37 °C for 17.5 h, the terminal amines were blocked and labelled with 8-plex iTRAQ reagents per the manufacturer’s protocol (Sciex). Following completion of the labelling protocol, the peptides were lyophilized using a centrifugal vacuum concentrator (Speed-Vac, Thermo Scientific). The experiment was performed in duplicate with the following iTRAP labelling: label 113 – Pro replicate 1, label 114 – ProPol replicate 1, label 115 – ppdko replicate 1, label 116 – Pro replicate 2, label 117 – ProPol replicate 2 and label 118 – ppdko replicate 2.

Desalting by solid-phase extraction was performed using Sep-Pak C18 cartridges (Waters) as described in [31]. The labelled peptides were analysed by nanoflow uHPLC coupled to a nano-spray source of a 5600+ TripleTOF mass spectrometer (AB Sciex). Therefore, the peptides were separated on an emitter-tip column with a gradient increasing from 5% ACN (v/v), 0.1% FA (v/v) to 45% ACN (v/v), 0.1% FA (v/v) to 95% ACN (v/v) and 0.1% FA (v/v) in water, followed by column washing at 98% ACN and re-equilibration at 5% ACN. The sample was injected in four technical replicates, each running separate liquid chromatography gradients (90, 120 and 2 times 150 min) at 400 nl min^−1^. Over the entire gradient time, the mass spectrometer acquired 1 precursor ion scan followed by 20 fragment ion scans for the 90 and 120 min methods or 30 fragment ion scans for the 150 min method, per acquisition cycle. The fragmentation energy was adjusted for the iTRAQ reporter ion detection.

Results were searched against a murine proteome database with the MNV-1.CW3 NS1-2 sequence added using Proteome Discoverer v.2.2 software (Thermo Scientific). Peptide identification parameters included semi-tryptic peptides with a maximum of two missed cleavage sites, the precursor ion and fragment mass tolerances set to 0.1 Da and the false discovery rate of <1%. The iTRAQ quantification node calculated the peptide abundance ratios based on the iTRAQ reporter ion intensities. The abundance ratios of the NS1-2 peptides were calculated by dividing the peptide abundance of the ProPol- or Pro-treated sample by the ppdko-treated sample. MS data can be accessed at ProteomeXchange with identifier PXD074184.

### Site-directed mutagenesis of MNV-1

To introduce mutations E66A, E66D and the double mutation E66D, L69H within the NS1-2 region of MNV-1 (GenBank DQ285629), primers were designed (Table 2) based on the protocol of Liu and Naismith [32].

**Table 2.** Mutagenesis primers used in this study

Name	Sequence 5′−3′^*^
NS1-2 E66D L69H F	GCGCCCTTGCGGCCCTTCATGCGGACGGGCCCCATGCC
NS1-2 E66D L69H R	GCATCACTACGCGTCACGGGGAGCCCGGCATGGGGCCCGTCCG
NS1-2 E66D F	GCGCCCTTGCGGCCCTTCATGCGGATGGGCCCCTTGCCG
NS1-2 E66D R	TGCATCACTACGCGTCACGGGGAGCCCGGCAAGGGGCCCATCC
NS1-2 E66A F	GGCGCCCTTGCGGCCCTTCATGCGGCAGGGCCCCT
NS1-2 E66A R	CGCGTCACGGGGAGCCCGGCAAGGGGCCCTGCCGC

*Underlined nucleotides indicate mutations and include the ApaI restriction site GGGCCC.

The previously described MNV-1 full-length cDNA clone [25] was used for mutagenesis. Briefly, primer pairs with an overlapping region containing the mutation of interest, as well as a non-complementary region binding to the template, were synthesized by IDT (Table 2). Mutagenesis PCRs were performed with Phusion high-fidelity polymerase [New England Biolabs (NEB)] according to the manufacturer’s protocol for templates larger than 10 kbp. The PCR products were gel-purified, then digested with 0.5 U of DpnI (Roche) at 37 °C for 2 h. The digested DNA was transformed into calcium-competent XL1-Blue *E. coli*. Clones were screened via ApaI (Roche) restriction digests, and the final plasmids were confirmed by whole-plasmid sequencing performed by Plasmidsaurus.

### Generation of recombinant virus

Recombinant MNV was generated according to [33]. Briefly, pMNV* was linearized with 2 U of NotI (NEB) and subjected to phenol/chloroform extraction and ethanol precipitation. Messenger RNA was transcribed from 1 µg of linearized DNA using the mMessage mMachine T7 Ultra kit (Thermo Scientific), including polyA tail addition, and the RNA was purified via the MEGAclear transcription cleanup kit (Thermo Scientific) according to the manufacturer’s protocol.

Huh7CD300lf cells were seeded at 3.5×10^5^ cells/well in a 6-well plate and transfected with 1.5 µg of MNV RNA using Turbofect (Thermo Scientific) per the manufacturer’s protocol. Briefly, 1.5 µg of RNA was incubated with 6 µl of Turbofect in 400 µl DMEM low glucose for 20 min at room temperature. The transfection reactions were added onto the cells. The cell supernatants were harvested 3 days post-transfection and stored at −80 °C. The viruses were passaged in Huh7CD300lf cells and purified at passage four as described above.

Samples were collected from the passage four supernatants, and viral RNA was extracted using the QIAamp viral RNA kit (Qiagen). cDNA was generated using Superscript III reverse transcriptase (Thermo Scientific) and the primers described in Table 3. The first-strand cDNA was amplified via two-step PCR, and the product sequence was confirmed by nanopore sequencing (Plasmidsaurus).

**Table 3.** Primers used to sequence MNV

Name	Sequence 5′−3′
MNV Full F	GTGAAATGAGGATGGCAACGCCATCT
MNV Full R	TTTTTTTTTTTTTTTTTTTTTTTTTTTTTTAAAATGC

### Statistical analyses

Statistical analysis was performed in GraphPad Prism 10. Significance between multiple groups was determined via one-way ANOVA with a Tukey or Bonferroni multiple comparisons test. The significance between the two groups was determined via an unpaired t-test. *P* values of less than 0.05 were considered significant.

## Results

### Identification of a partially conserved viral protease site in MNV-1 NS1-2

**Fig. 1.**
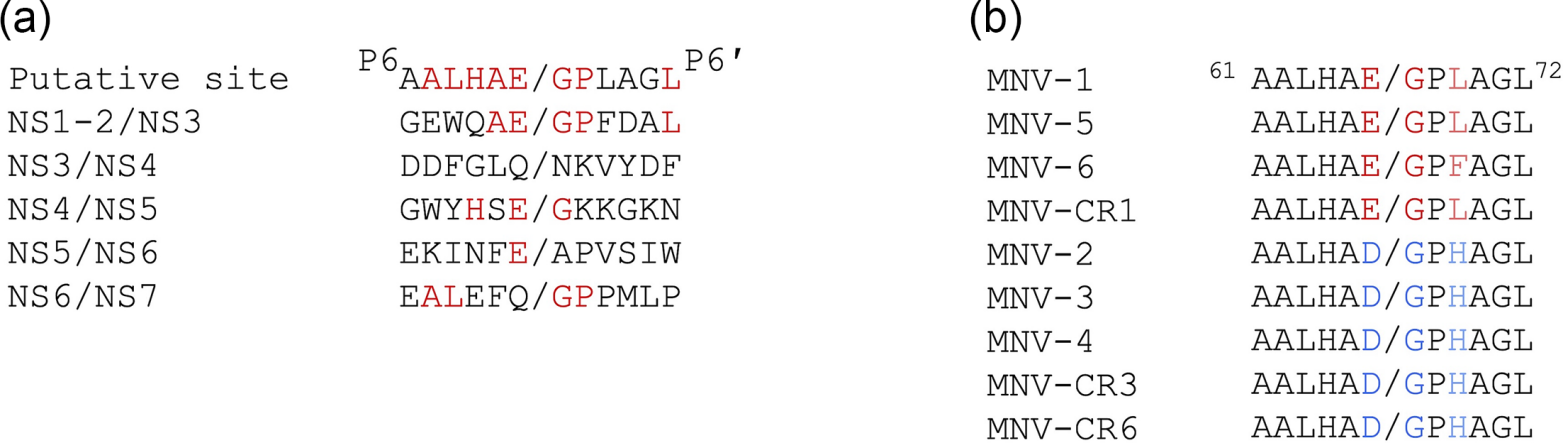
Identification of a partially conserved protease site in the NS1 region of MNV-1 NS1-2. (**a**) Alignment of the P6-P6′ residues of the putative viral protease site to the established ORF1 NS6 cleavage sites (DQ285629). Amino acids conserved between the putative NS6 protease site and the established sites are coloured red. (**b**) Amino acids 61–72 (P6-P6′) were aligned from MNV-1 DQ285629, MNV-2 ABB02416.1, MNV-3 ABB02419.1, MNV-4 DQ223043, MNV-5 ABS29272.1, MNV-6 EF650481.1, MNV-CR1 EU004672, MNV-CR3 EU004673.1 and MNV-CR6 EU004676. The two core motifs are highlighted in red (E/GPL) or blue (D/GPH). Scissile bonds are denoted by a slash.

Multiple fragments of MNV-1 NS1-2 are observed by Western blotting during infection [22], including a 36 kDa fragment (herein designated NS1c-2) that must be produced independently of caspase cleavage at amino acid 121 [21]. The MNV-1 NS1-2 sequence was analysed to identify putative viral protease sites that could generate the NS1c-2 protein upon cleavage. A potential viral protease cleavage site was identified between E^66^ and G^67^ in the NS1 region of NS1-2 that would be consistent with the production of a 36 kDa fragment. The P6-P6' amino acids of the putative protease site were compared to the corresponding amino acids of each of the characterized ORF1 protease cleavage sites (Fig. 1a), with the P5-P2′ amino acids found to be conserved in at least one other established cleavage site. Furthermore, the P2-P2′ residues (AEGP) known to have a major role in determining norovirus protease site recognition [34] were conserved with the NS1-2/NS3 cleavage site.

Nine MNV strains were aligned to determine if the putative E^66^/G^67^ protease site was conserved (Fig. 1b). The alignment showed that E^66^ was conserved in multiple MNV strains and contained either a hydrophobic leucine or phenylalanine at position 69, mimicking the MNV NS1-2/NS3 cleavage site. A second clade of MNV strains was identified with an aspartic acid at position 66, paired with a histidine at position 69, which may prevent the viral protease from cleaving at this site as D/G has not been identified as a norovirus protease site. These results indicate that MNV NS1 sequences at this point in the protein divide into two clades: strains represented by E/GPL(F) that mimic a norovirus NS6 protease cleavage site and strains represented by D/GPH that do not mimic NS6 protease cleavage sites.

While the DGPH motif is unlikely to be a viral protease site, we investigated whether this could be a caspase cleavage site using ProsperousPlus software [28]. The known caspase sites at 121 and 131 [21] were strongly predicted to be cleaved; however, while no or limited cleavage was predicted at the E^66^/G^67^ site in the AEGP MNV-1 motif, analysis of the CR6 strain indicated a high likelihood of processing by caspases at the D^66^/G^67^ site in the DGPH MNV-CR6 motif (Table 4). While this study focuses on viral Pro vs ProPol specificity, cleavage at this position is likely to be a highly conserved event, albeit by variable mechanisms.

**Table 4.** Caspase cleavage prediction of NS1 for MNV-1 and CR6 strains

	MNV-1			MNV-CR6		
	LHAE^66^/GP	DRPD^121^/AP	DAMD^131^/AK	LHAD^66^/GP	DKAD^121^/AP	DAMD^131^/AR
Casp 3	0.013^*^	1.000	0.970	0.985	0.995	0.986
Casp 6	0.014	0.758	0.969	0.855	0.971	0.949
Casp 7	0.675	0.964	0.982	0.953	0.978	0.978
Casp 8	0.484	0.991	0.998	0.998	0.998	0.997

*ProsperousPlus [28] prediction of caspase processing. Scores below a 0.75 cut-off are shaded.

**Fig. 2.**
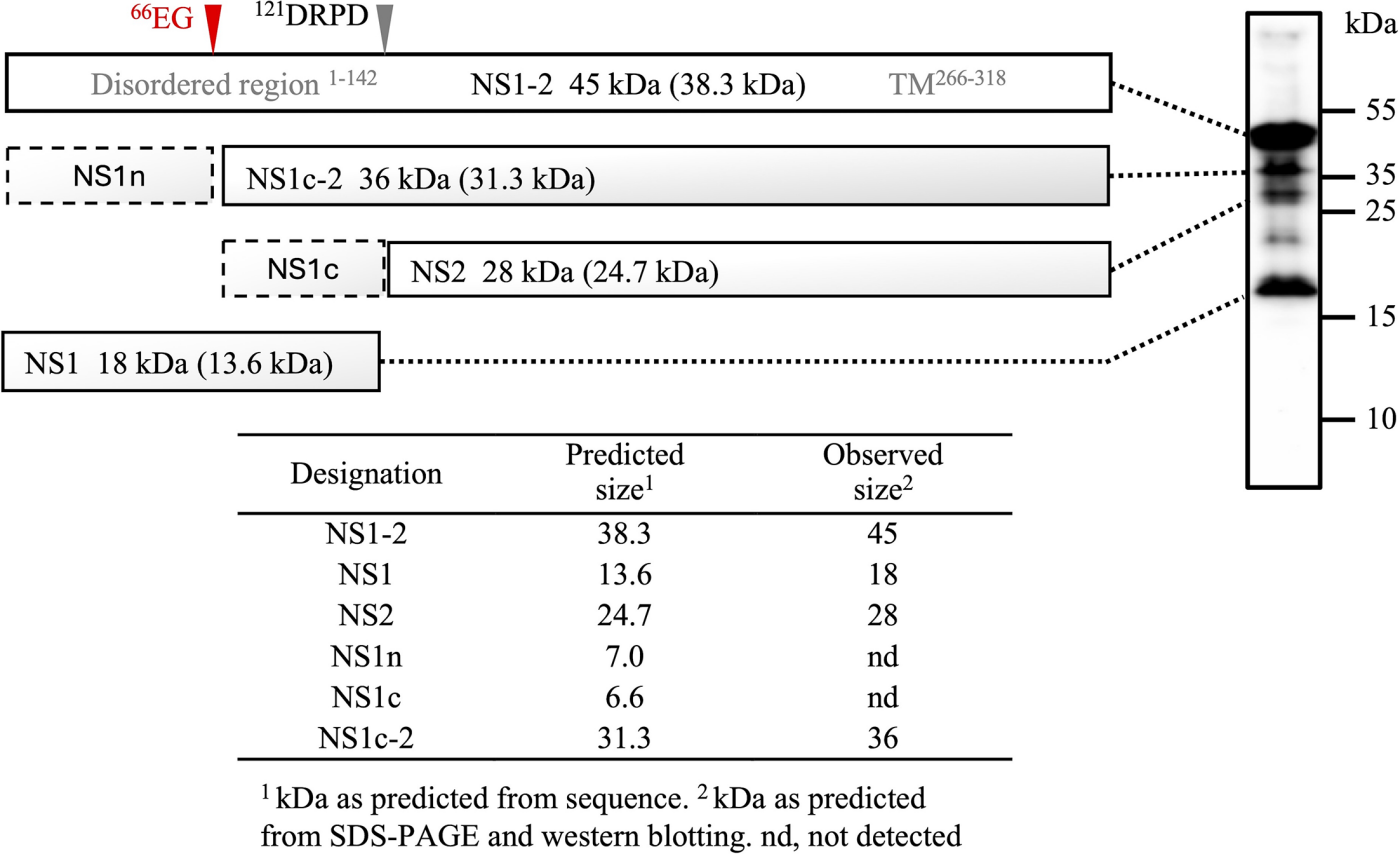
Proposed viral protease site in MNV-1 NS1-2. Schematic of MNV-1 NS1-2 with the proposed identity and naming designation of each fragment. A grey arrow indicates the known caspase cleavage site, and the red arrow indicates the proposed ^66^EG viral protease site. NS1 and NS2 are designated as caspase cleavage products at the DRPD site. The N-terminal region of NS1 (dashed box), designated NS1n, was not detected. NS1-2 cleaved by MNV-1 NS6 (Pro) but not caspases, comprising the C-terminal region of NS1 and all of NS2, was designated NS1c-2. Observed fragment sizes by SDS-PAGE analysis are indicated with predicted sizes from the amino acid sequence shown in brackets. A summary of predicted vs observed sizes for each designated fragment is shown. NS1-2 fragments are mapped by dotted lines to Western blot bands of total MNV-1-infected RAW264.7 cell lysate probed with an anti-NS1-2 polyclonal antibody. Molecular weight marker sizes are indicated. The Western blot is adapted from [22].

Noting that disordered NS1 migrates slower than expected for its molecular weight on SDS-PAGE gels [22], cleavage between E^66^ and G^67^ would generate fragments of 7 kDa (herein designated NS1n) and 31 kDa (NS1c-2). The NS1n fragment was not detected. The predicted size of NS1c-2 at 31 kDa is consistent with an estimated size of 36 kDa when visualized by SDS-PAGE (Fig. 2).

### The protease precursor ProPol cleaves between E^66^ and G^67^ in MNV-1 NS1-2

MNV ProPol, Pro and ppdko with native N- and C-termini were expressed and purified from *E. coli* using a SUMO fusion cloning system (Lucigen). The protease activity of the purified recombinant ProPol and Pro was confirmed on a fluorometric peptide designed from the ORF1 NS1-2/NS3 cleavage site. Both ProPol and Pro were active on the peptide substrate, and the data were fitted to a Michaelis–Menten model. MNV ProPol and Pro had similar affinities to the NS1-2/NS3 substrate as estimated by Km at 67.4±8.7 and 63.2±2.6 µM, respectively. In contrast, ProPol demonstrated a higher Kcat at 110.6±11.5 vs 85.36±8.7 s^−1^ (*P*≤0.05) and catalytic efficiency (Kcat/Km) of 1.8±0.2 vs 1.3±0.1 (*P*≤0.05) than Pro.

**Fig. 3.**
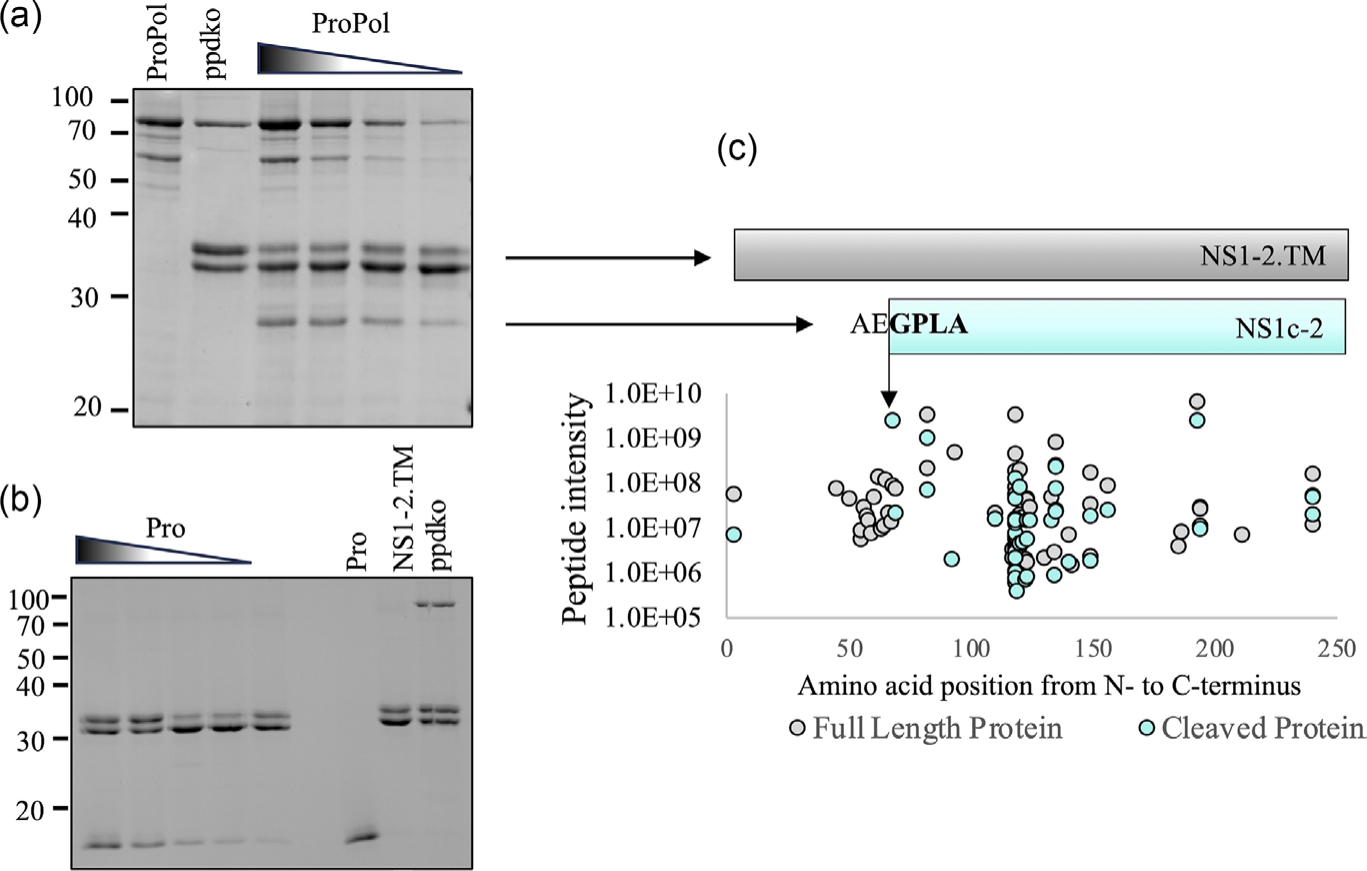
MNV NS1-2.TM is cleaved by MNV ProPol but not Pro. MNV NS1-2.TM (7.5 µM) was incubated with MNV ProPol (**a**) or Pro (**b**) at concentrations varying from 6.0 to 0.75 or 6.0–0.37 µM, respectively, at 37 °C for 4.5 h. Purified ProPol is shown in (**a**), and purified Pro and NS1-2.TM (doublet) are shown in (**b**). Inactive ProPol ppdko incubation with NS1-2.TM is shown on both gels. (**c**) The cleaved NS1c-2-TM fragment and uncleaved NS1-2.TM in (a) were excised from the SDS-PAGE gel, and the amino acid sequences were determined by LC-MS (**c**). The intensities of the peptides identified in both proteins were plotted against the amino acid position from the N- to C-terminus of NS1-2.TM. Peptides identified in NS1-2.TM are coloured grey, and peptides identified in the NS1c-2 fragment are coloured cyan.

To identify whether the putative NS1c-2 site is cleaved by either form of the viral protease in the context of a recombinant protein, MNV-1 NS1-2 with the insoluble transmembrane domain removed (NS1-2.TM; predicted size 29.1 kDa, observed size 32 kDa) was expressed and purified [20] and then titrated with varying concentrations of MNV ProPol (Fig. 3a) or Pro (Fig. 3b). A 25 kDa band consistent with cleavage at E^66^/G^67^ to generate NS1c-2.TM was observed when the NS1-2.TM was incubated with ProPol but not Pro, with cleavage decreasing upon titration of ProPol (Fig. 3a).

Analysis of uncleaved NS1-2.TM identified strong tryptic peptide signals covering the majority of the protein (Fig. 3c), including the sequences N-terminal to the proposed E/G cleavage site. Conversely, in NS1c-2, the near-total absence of strong peptides signals N-terminal of G^67^ combined with the detection of the specific peptide ^67^GPLAGLPVTR^76^ indicates that E^66^/G^67^ is the scissile bond cleaved by ProPol. The one peptide identified from the cleaved protein sample N-terminal of the cleavage site (cyan dot) is attributed to a fragment of NS1-2 running in the same gel lane.

To confirm cleavage of NS1-2.TM by ProPol or Pro in a complex environment RAW264.7, cell lysates spiked with 5 µg NS1-2.TM were incubated with ProPol, Pro or ppdko and labelled with isobaric tags to identify the N-terminal peptides generated by the enzyme digestion using the TAILS method. The ^67^GPLAGLPVTR^76^ peptide indicative of cleavage at E^66^/G^67^ was detected at a 4.9- and 4.6-fold higher amount in both ProPol replicates versus the respective ppdko controls, whereas the Pro-treated samples only showed a minor increase of 1.8- and 1.4-fold compared to the ppdko control (a reference spectrum for the identification of the N-terminal peptide is shown in Fig. S1, available in the online Supplementary Material).

### Cleavage at E^66^/G^67^ occurs during MNV infection

**Fig. 4.**
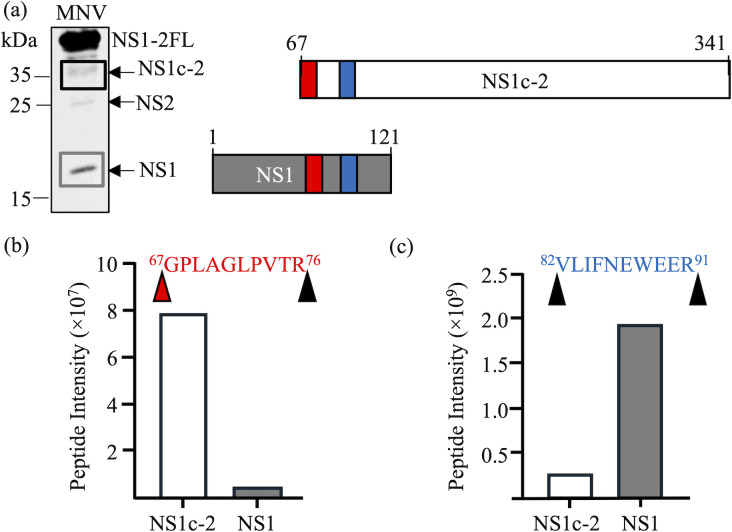
ProPol processing of NS1-2 occurs during MNV-1 infection. (**a**) BV-2 cells were infected at an MOI of 10. Samples were harvested at 18 hpi, and the Western blot was performed with an anti-NS1-2 antibody. Two regions of a matching SDS-PAGE gel representing NS1c-2 and NS1 were excised (black and grey boxes). The location of targeted peptides ^67^GPLAGLPVTR^76^ (red box) and ^82^VLIFNEWEER^91^ (blue box) is shown. (**b**) The peptide intensity of the ^67^GPLAGLPVTR^76^ peptide, as determined by MS, that requires ProPol cleavage is shown for NS1 and NS1c-2. (**c**) The peptide intensity of the trypsin-generated peptide ^82^VLIFNEWEER^91^ detected in NS1 and NS1c-2 is shown. Black arrowheads indicate trypsin cleavage sites, and the red arrowhead indicates the ProPol cleavage site.

To determine if cleavage between E^66^ and G^67^ in NS1-2 occurred during infection, BV-2 cells were infected with MNV and harvested at 18 h post-infection (hpi). NS1c-2 and NS1 fragments detected via Western blot (Fig. 4a) were excised and analysed by MS.

Two peptides were quantified by MS from the NS1 and NS1c-2 fragments (Fig. 4), with ^82^VLIFNEWEER^91^ being a tryptic peptide generated during sample preparation that is present in both NS1 and NS1c-2, thereby serving as an internal reference for protein abundance (Fig. 4c). The peptide ^67^GPLAGLPVTR^76^ corresponds to the N-terminus of the NS1c-2 fragment consistent with ProPol cleavage at the E^66^/G^67^ site (reference spectrum is shown in Fig. S2); furthermore, this peptide is at very low abundance in the NS1 fragment relative to the NS1c-2 fragment, despite NS1 being the more abundant species (Fig. 4b). Combined, these data confirm that NS1c2 is generated during MNV infection.

### Processing at E^66^/G^67^ is important for viral replication

**Fig. 5.**
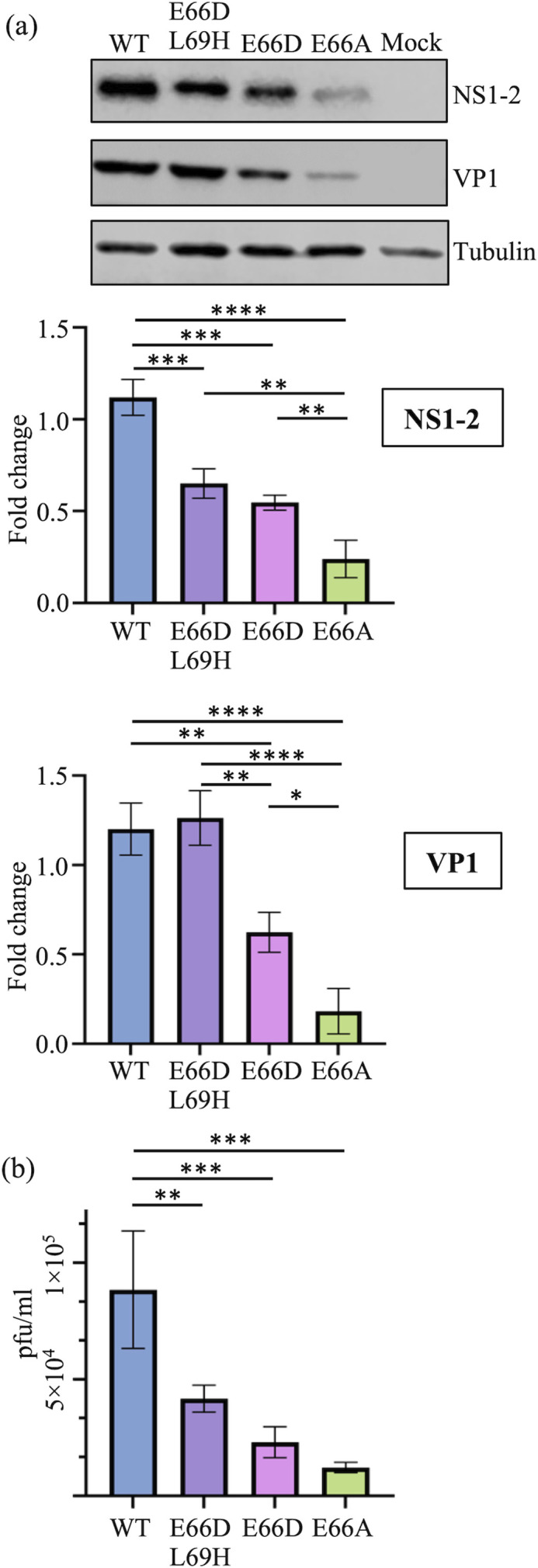
Mutations in the NS1c-2 ProPol cleavage site impact MNV-1 replication. Huh7CD300lf cells were infected with MNV-1 WT, MNV-1 E66D L69H, MNV-1 E66D or MNV-1 E66A at an MOI of 0.1. Cells were harvested at 12 hpi. (**a**) Samples were visualized via Western blot with anti-NS1-2 or anti-VP1 antibodies with tubulin antibodies as a loading control. Quantification of viral proteins from three independent experiments was performed in ImageStudio with band signals normalized to tubulin and then displayed relative to a WT control. Statistical significance was determined via one-way ANOVA using Tukey’s multiple comparisons test, and data are presented as mean±sd. *=*P*≤0.05, **=*P*≤0.005, ***=*P*≤0.0005 and ****=*P*≤0.00005. (**b**) Cell culture supernatant was harvested at 12 hpi. Virus titre was determined via plaque assay in biological triplicate. The data shown are mean±sd from three independent experiments. Statistical significance was determined via one-way ANOVA. **=*P*≤0.005, ***=*P*≤0.005.

Recombinant MNV carrying mutations E66D to generate a D/G cleavage site, E66A as a non-processible control or the double mutant E66D and L69H to mimic the DGPH clade (Fig. 1b) were generated. Huh7CD300lf cells containing the murine CD300lf receptor were infected with MNV-1 WT, MNV-1 E66D-L69H, MNV-1 E66D or MNV-1 E66A viruses at an MOI of 0.1. The level of NS1-2 observed by Western blotting (Fig. 5a) was reduced in all the mutant viruses compared to MNV-1 WT. The expression of VP1 was also reduced for E66D and E66A viruses; however, VP1 levels were near that of the WT virus for the E66D-L69H virus representing the DGPH clade motif mutation that was predicted to be a caspase cleavage site, suggesting that cleavage at this site may be important for all MNV strains. The E66A virus displayed the largest decrease in both NS1-2 and VP1 expression, confirming that processing of NS1-2 by ProPol is important for viral protein expression.

Extracellular virus titres at 12 hpi were determined via plaque assay (Fig. 5b). All of the NS1-2 mutant viruses showed a reduction in virus titre compared to the MNV-1 WT virus commensurate with reduced levels of NS1-2 and/or VP1 in Fig. 5. Taken together, mutations to the ProPol cleavage site in the NS1-2 protein of MNV-1 reduced viral replication in Huh7CD300lf cells.

### Cleavage between E^66^ and G^67^ is critical for replication in a murine cell line

Because mutations to NS1-2 influence the adaptation of MNV to replicate in a human cell line [35] and cleavage of MNV strain CR6 NS1-2 by cellular caspases is important for infection of intestinal epithelial cells in mice [21], we hypothesized that the replication of the mutant viruses could be more or less impaired in a naturally permissive murine cell line.

**Fig. 6.**
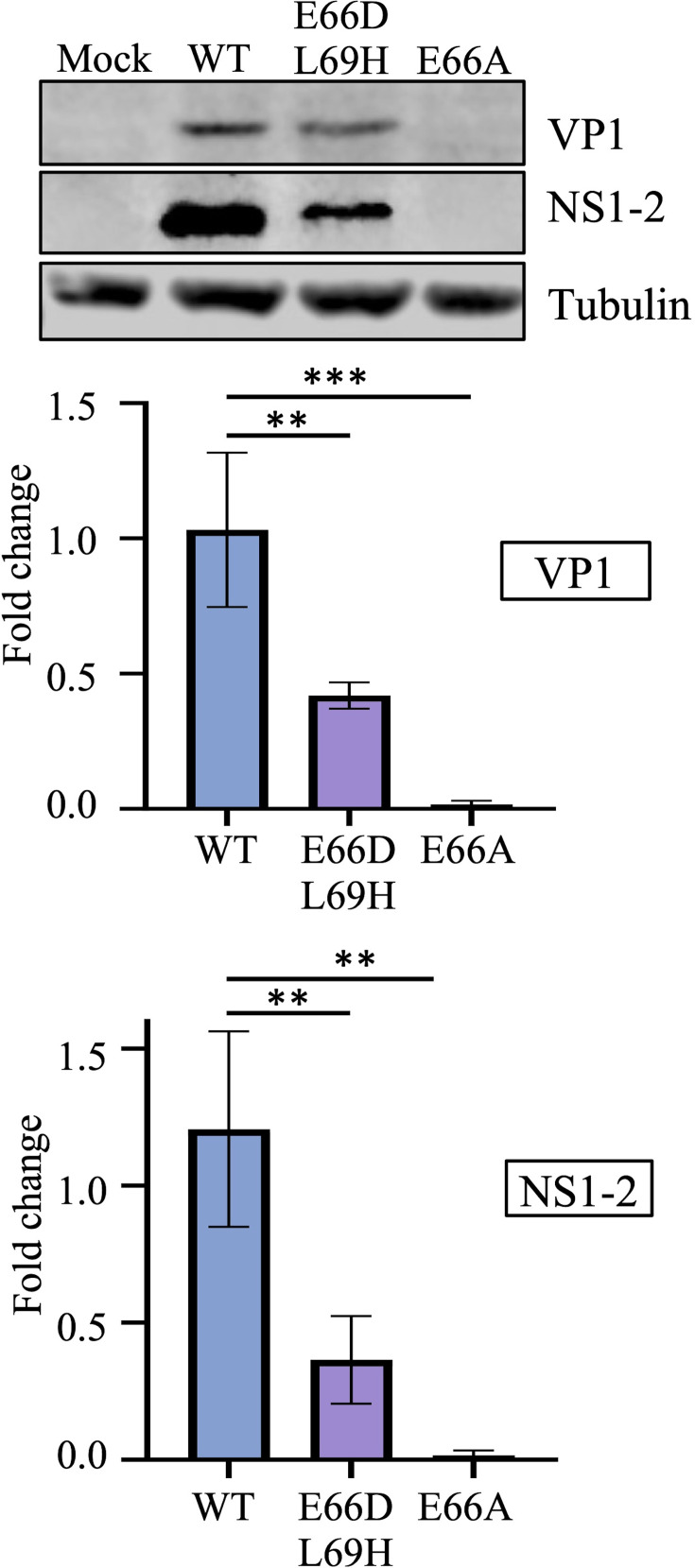
Mutations to the NS1c-2 ProPol cleavage site reduce viral protein expression in a murine cell line. BV-2 cells were infected with MNV-1 WT, MNV-1 E66D L69H or MNV-1 E66A at an MOI of 0.1. Cells were harvested at 12 hpi, and samples were visualized via Western blot with anti-NS1-2 or anti-VP1 antibodies. Tubulin was used as a loading control. Quantification of NS1-2 and VP1 was performed in ImageStudio with band signals normalized to tubulin and displayed relative to a WT control. Statistical significance was determined via one-way ANOVA using Tukey’s multiple comparisons test. Data are presented as mean±sd (*n*=3) for independent experiments. **=*P*>0.005, ***=*P*>0.0005 and ****=*P*>0.00005.

BV-2 cells were infected with MNV-1 WT, MNV-1 E66D L69H or MNV-1 E66A viruses at an MOI of 0.1 and incubated for 12 h. Expression of NS1-2 was reduced for mutant viruses to a similar level as seen in Huh7CD300lf cells (Fig. 6). In contrast, VP1 expression was significantly lower in the BV2 cells with the MNV-1 E66D L69H mutant, approximately threefold less than the MNV-1 WT type virus (Fig. 6). Furthermore, the E66A mutation led to almost undetectable expression of NS1-2 and VP1 in the murine cell line.

**Fig. 7.**
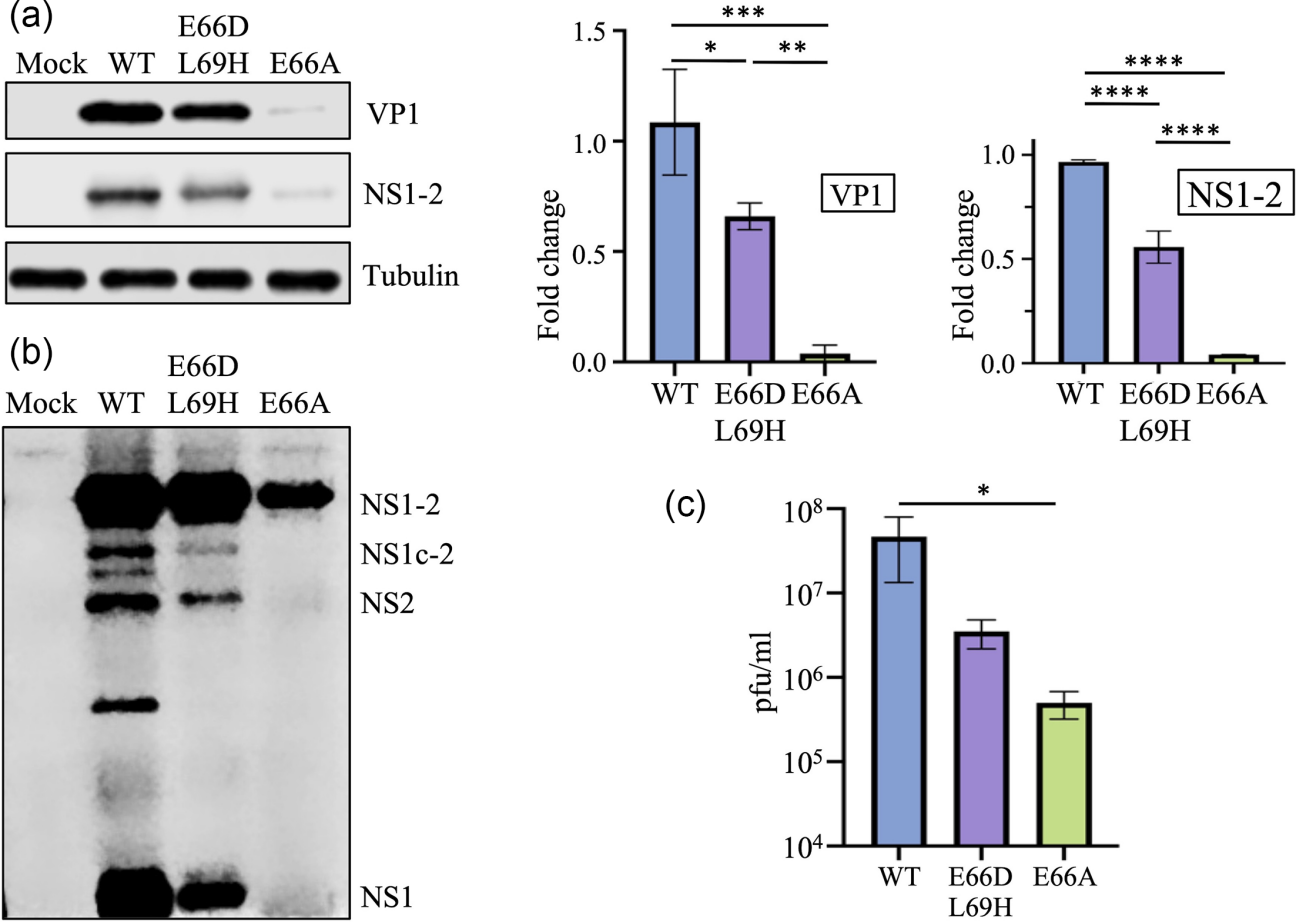
Mutations to the NS1c-2 ProPol cleavage site reduced viral replication in a murine cell line. BV-2 cells were infected with MNV-1 WT, MNV-1 E66D L69H or MNV-1 E66A at an MOI of 3. Cells and cell supernatant were harvested at 18 hpi, and the samples were visualized via Western blot with anti-NS1-2 or anti-VP1 with tubulin used as a loading control. Quantification of viral proteins was performed in ImageStudio with band signals normalized to a WT control. (**b**) Overexposure of the NS1-2 blot from (a). (**c**) The virus titre was determined via plaque assay in BV-2 cells. Statistical significance for (a) and (c) was determined via one-way ANOVA using Tukey’s multiple comparisons test, and data are presented as mean±sd from three independent experiments. *=*P*>0.05, **=*P*>0.005, ***=*P*>0.0005 and ****=*P*>0.00005.

The replication of the MNV-1 E66D L69H and MNV-1 E66A mutants in the BV-2 cells was repeated to determine if a higher MOI of 3.0 and an 18 h incubation period could rescue replication in BV2 cells. The reduction in NS1-2 and VP1 for MNV-1 E66D L69H and E66A (Fig. 7a) was similar to that observed at the lower MOI and shorter incubation period (Fig. 6), albeit the reduction in expression was less for the E66D L69H virus at an approximately twofold reduction. The MNV-1 E66A mutant had the largest reduction in NS1-2 and VP1 expression (~25-fold). Overexposure of the blot from Fig. 7a to generate Fig. 7b showed substantially reduced cleavage at the E^66^/G^67^ target site, including no detectable cleavage of E66A to produce NS1c-2. Importantly, the virus titre of the E66D L69H virus in cell culture medium was reduced ~10-fold, and the E66A mutant was decreased by ~100-fold (Fig. 7c). Overall, mutations to the ProPol cleavage site in MNV-1 NS1-2 are detrimental to virus replication.

## Discussion

**Fig. 8.**
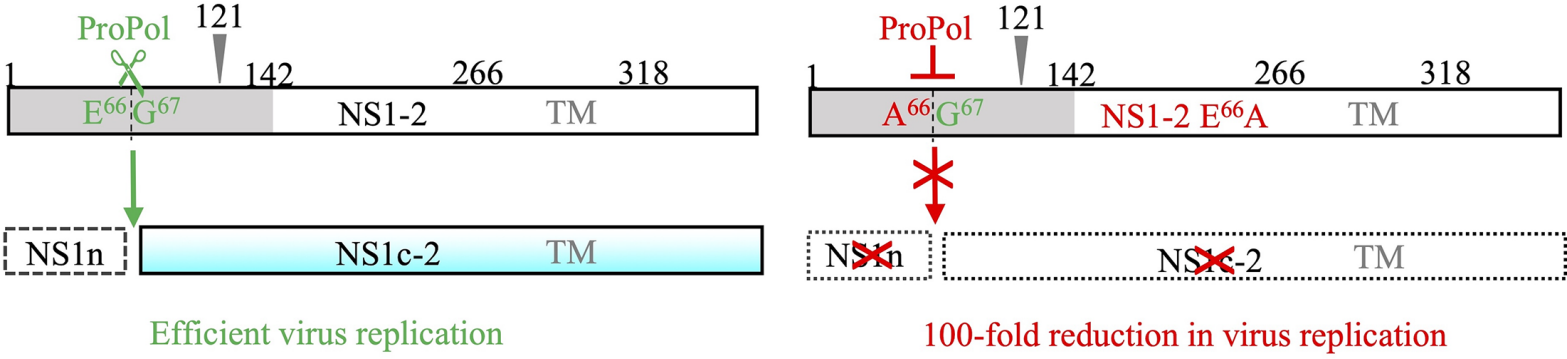
A schematic diagram of ProPol cleavage of MNV-1 NS1-2 during infection. NS1-2 is cleaved from the ORF1 polyprotein during MNV-1 infection. ProPol recognizes and cleaves the E^66^ protease site to produce NS1n and NS1c-2. When the ProPol cleavage site is mutated (E^66^A), ProPol does not cleave NS1-2, thereby preventing production of NS1c-2 and undetected potential fragments such as NS1n. The modification of the site to prevent detectable cleavage reduced viral titres ~100-fold. The caspase cleavage site at position 121 that generates NS1 and NS2 is indicated by a grey arrowhead. The disordered region from 1 to 142 is in grey. TM, transmembrane region 266–318.

The role of the protease function of ProPol in MNV-1 infection is only partially understood. In this study, we characterized a previously undescribed protease site in the NS1-2 region of the ORF1 polyprotein (Fig. 8) that is cleaved by ProPol but not Pro to affect MNV-1 replication in multiple cell lines, confirming that ProPol protease can target distinct substrates to Pro that are important for the replication of MNV-1.

Prediction of the protein fragments produced from cleavage at the E^66^/G^67^ site corresponded to a previously undescribed fragment designated NS1c-2 observed during MNV infection that had previously been proposed to be due to an alternative translational start or host protease cleavage [21,22]. However, the amino acid sequence of the putative protease site resembled the established MNV-1 NS1-2/NS3 protease site and also resembles the NS1/NS2 protease sites in sapoviruses and vesiviruses [12,36] and, to a lesser extent, RHDV (lagovirus) [11]. In each of these viruses, the ProPol precursor was identified as the main protease [12] or as an abundant precursor [11,36], and this study confirms an important role for the ProPol protease of a norovirus that is distinct to the mature Pro.

MS indicated that the N-terminus of the NS1c-2.TM fragment generated by ProPol was the predicted G67 amino acid, and this was confirmed by the identification of the G^67^-R^76^ peptide in the ProPol-treated lysate. This peptide was also identified in an MNV-1-infected sample, confirming that this processing was not an artefact of off-target effects in an *in vitro* assay.

Consistent with a biologically important event, the cleavage site is conserved among MNV strains in two forms, with the EGPL or the DGPH variants mapping to the prototypical acute/persistent viruses MNV-1 and MNV-CR6, respectively. It is possible to speculate that acute strains are processed by the early ProPol protein (EGPL clade), and persistent strains are processed by caspases stimulated later in infection (DGPH clade), but correlation of the EGPL and DGPH variants to other acute or persistent MNV strains is not complete [37]. However, not all MNV strains cleanly fit these phenotypes (MNV-S66 and MNV-WU11) [38,39], and the ProPol cleavage site may influence the replication of these strains.

In order to determine if processing of NS1-2 by ProPol was important for viral replication, a series of three mutant viruses was generated. The E66A mutation had the greatest decrease in both viral protein expression and titre in the Huh7CD300lf cells. In addition to inhibiting ProPol processing, the E66A mutation was also designed to prevent cellular caspase cleavage.

The replication of the MNV-1 E66D L69H virus was the most comparable to the WT virus, although a decrease in NS1-2 expression and viral titre was still observed. Previous comparisons of the growth kinetics between viruses with E^66^ and D^66^ sequence variants identified no difference in cell culture [37]. However, domain swaps substituting the D^66^ NS1 (CR6) into an E^66^ virus backbone (CW1) showed a delay in virus egress [40]. Analysis of the DGPH strains predicts a LHAD/GPHA caspase cleavage site [41] rather than a ProPol processing site that may influence virus replication rate, although there is no experimental evidence for this hypothesis.

The E66A mutation should be an inefficient site for both ProPol and caspases, as confirmed in Fig. 7. Analysis of the viral protein expression and titre in BV-2 cells at low and high MOI (0.1 and 3, respectively) showed that the E66A mutation to the ProPol cleavage site decreased viral protein expression and caused a 100-fold decline in viral titre in this naturally permissive murine cell line, indicating that cleavage of NS1-2 at this site is important for replication.

While we tracked the NS1c-2 fragment, a number of different fragments would be produced from combinations of caspase and ProPol cleavage of NS1-2. Like many other viruses, it seems likely that the various fragments will have an as-yet undetermined role in virus replication, and which, if any, of these caused the decline in protein production and viral replication is unknown.

The mechanisms by which NS1 cleavage affects viral replication were not explored in this study. However, it was reported that MNV-CR6 NS1 binds to phosphatidylinositol phosphate (PIP) lipids and that the binding affinity is reduced when residues 1–64 are truncated [42]. PIP lipids have been implicated in the lifecycle of multiple positive-sense RNA viruses, with the interaction of poliovirus 3CD with PIPs proposed to facilitate sequestration of phospholipids [43], while phosphatidylinositol-3-phosphate is important in forming the tombusvirus replication compartment [44].

While we did not detect NS1n in our assays, the E^66^/G^67^ cleavage site is close to the FFAT motif (core residues Y47–Q53) that interacts with the host VAMP-associated protein A (VAPA) [45]. ProPol release of NS1n may increase accessibility to the FFAT motif, thereby allowing the binding of NS1n to VAPA and/or VAPB.

It is interesting to predict possible roles for the NS1c and NS1n fragments; however, we also note that the NS1c-2 protein identified during viral replication represents a form of NS2 that would be present early in infection prior to caspase induction. This modified form of NS2 may be the important protein product from cleavage of the NS1/2 protein. Complementation studies on the mutant viruses might provide a means to address the importance of NS1c, NS1n or NS1c-2.

In summary, this work shows that MNV-1 ProPol has protease activity distinct to mature Pro in recognizing a previously undescribed ProPol protease site in the MNV-1 NS1-2 protein. Cleavage at this site occurs during virus infection, and mutations that disrupted ProPol cleavage of NS1-2 were detrimental to MNV replication. Furthermore, MNV strains that do not have this ProPol cleavage site are strongly predicted to have a caspase site. Our results indicate that the protease domain of MNV-1 ProPol and its NS1 target site are important during MNV-1 replication.

## Supplementary material

10.1099/jgv.0.002263 Uncited Supplementary Material 1.

## Abbreviations

ACN: acetonitrile
DDA-MS/MS: data-dependent acquisition tandem MS
DMEM: Dulbecco’s modified Eagle’s medium
FA: formic acid
hpi: hours post-infection
HuNVs: human noroviruses
IDT: Integrated DNA Technologies
MNV: murine norovirus
NEB: New England Biolabs
NS: non-structural
PIP: phosphatidylinositol phosphate
PRM: parallel reaction monitoring
ProPol: protease–polymerase
uHPLC: ultra HPLC
VAPA: VAMP-associated protein A
