# Supplementary material for: The MNV-1 protease–polymerase precursor cleaves a novel site in the NS1-2 protein
Source: J Gen Virol. 2026 Apr 29;107(4):002263. doi: 10.1099/jgv.0.002263 (PMC13135481; doi:10.1099/jgv.0.002263)
Supplement: Uncited Supplementary Material 1. [file jgv-107-02263-s001.pdf]

Supplementary Material:

The MNV-1 ProPol precursor Cleaves a Novel Site in the NS1-2 Protein

Vernon K. Ward[1,4*], Geena M. McKenzie-Goldsmith[1], Matt J. Edwards[1], Vivienne L. Young[1], Torsten Kleffmann[2], Louise A. Stubbing[3], Andrew Siow[3], Margaret A. Brimble[3,4].

[1] Department of Microbiology and Immunology, Faculty of Biomedical Sciences, 720 Cumberland St, University of Otago, PO Box 56, Dunedin 9054, New Zealand.
[2] Centre for Protein Research, Research Infrastructure Centre, Division of Health Sciences, University of Otago, Dunedin 9054, New Zealand.
[3] School of Chemical Sciences, The University of Auckland, 23 Symonds Street and School of Biological Sciences, 3b Symonds Street, Auckland 1142, New Zealand.
[4] Maurice Wilkins Centre for Molecular Biodiscovery, The University of Auckland, 3b Symonds Street, Auckland 1142, New Zealand.
* Corresponding author.

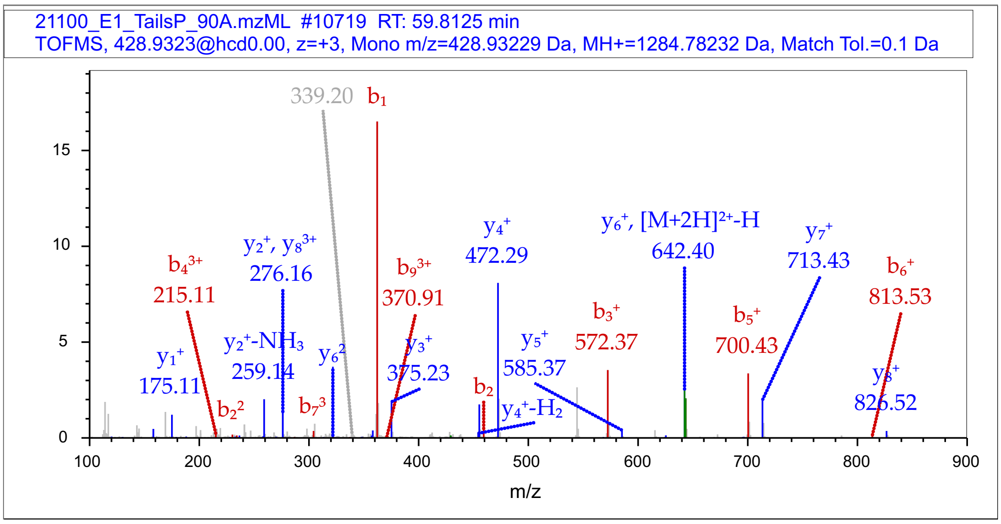

#1	b⁺	b²⁺	b³⁺	Seq.	y⁺	y²⁺	y³⁺	#2
1	362.23410	181.62069	121.41622	G-iTRAQ8...				10
2	459.28686	230.14707	153.76714	P	923.56728	462.28728	308.52728	9
3	572.37093	286.68910	191.46183	L	826.51451	413.76090	276.17636	8
4	643.40804	322.20766	215.14087	A	713.43045	357.21886	238.48167	7
5	700.42951	350.71839	234.14802	G	642.39334	321.70031	214.80263	6
6	813.51357	407.26042	271.84271	L	585.37187	293.18957	195.79548	5
7	910.56633	455.78680	304.19363	P	472.28781	236.64754	158.10079	4
8	1009.63475	505.32101	337.21643	V	375.23504	188.12116	125.74987	3
9	1110.68243	555.84485	370.89899	T	276.16663	138.58695	92.72706	2
10				R	175.11895	88.06311	59.04450	1

Supplemental Fig.1. Annotated spectra and fragment ion tables of the NS1c-2 N-terminal peptide [67]GPLAGLPVTR[76] detected as an iTRAQ-labelled triply charged precursor ion in the TAILS experiment by TripleTOF-MS Data are available via ProteomeXchange with identifier PXD074184.

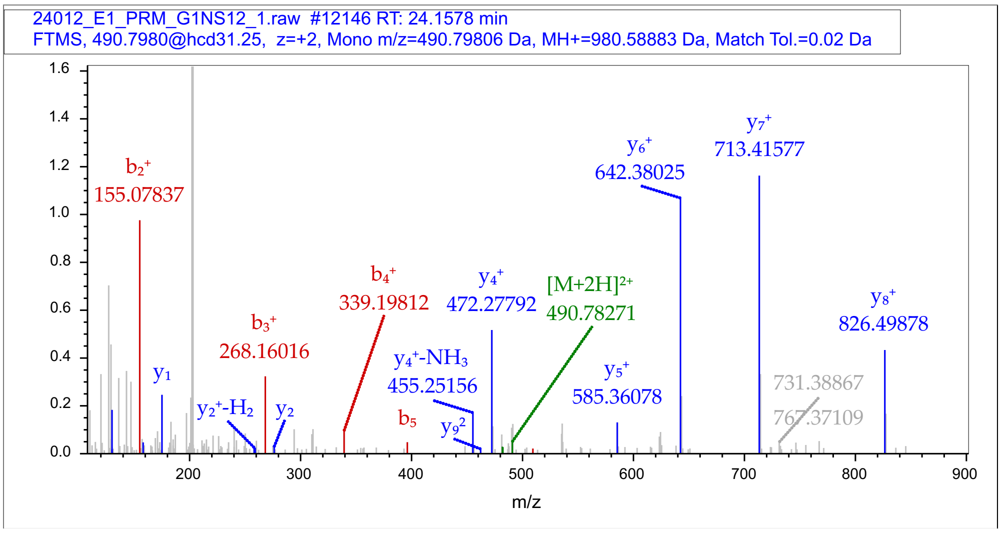

#1	a⁺	a²⁺	b⁺	b²⁺	Seq.	y⁺	y²⁺	#2
1	30.03383	15.52055	58.02874	29.51801	G			10
2	127.08659	64.04693	155.08150	78.04439	P	923.56728	462.28728	9
3	240.17065	120.58897	268.16557	134.58642	L	826.51451	413.76090	8
4	311.20777	156.10752	339.20268	170.10498	A	713.43045	357.21886	7
5	368.22923	184.61825	396.22415	198.61571	G	642.39334	321.70031	6
6	481.31329	241.16029	509.30821	255.15774	L	585.37187	293.18957	5
7	578.36606	289.68667	606.36097	303.68412	P	472.28781	236.64754	4
8	677.43447	339.22087	705.42939	353.21833	V	375.23504	188.12116	3
9	778.48215	389.74471	806.47707	403.74217	T	276.16663	138.58695	2
10					R	175.11895	88.06311	1

Supplemental Fig. 2. Annotated spectra and fragment ion tables of the NS1c-2 N-terminal peptide ^{67}GPLAGLPVTR^{76} detected as a doubly charged precursor ion targeted by parallel reaction monitoring using an Orbitrap Exploris 240 mass spectrometer. Data are available via ProteomeXchange with identifier PXD074184.

PRIDE database references:

Perez-Riverol Y, Bandla C, Kundu DJ, Kamatchinathan S, Bai J, Hewapathirana S, John NS, Prakash A, Walzer M, Wang S, Vizcaíno JA. The PRIDE database at 20 years: 2025 update. Nucleic Acids Res. 2025 Jan 6;53(D1):D543-D553. doi: 10.1093/nar/gkae1011. (PubMed ID: 39494541).

Deutsch EW, Bandeira N, Perez-Riverol Y, Sharma V, Carver JJ, Mendoza L, Kundu DJ, Bandla C, Kamatchinathan S, Hewapathirana S, Sun Z, Kawano S, Okuda S, Connolly B, MacLean B, MacCoss MJ, Chen T, Zhu Y, Ishihama Y, Vizcaíno JA. The ProteomeXchange consortium in 2026: making proteomics data FAIR. Nucleic Acids Res. 2025 Nov 6:gkaf1146. doi: 10.1093/nar/gkaf1146. Epub ahead of print. PMID: 41206473.

Perez-Riverol Y, Xu QW, Wang R, Uszkoreit J, Griss J, Sanchez A, Reisinger F, Csordas A, Ternent T, del Toro N, Dianes JA, Eisenacher M, Hermjakob H, Vizcaíno JA (2016). PRIDE Inspector Toolsuite: moving towards a universal visualization tool for proteomics data standard formats and quality assessment of ProteomeXchange datasets. Mol Cell Proteomics 15(1):305-17 (PubMed ID: 26545397).
